# Knowledge and Awareness of Parents Regarding Congenital Inguinal Hernia and Its Complications in the Pediatric Population in Makkah Region, Saudi Arabia

**DOI:** 10.7759/cureus.100120

**Published:** 2025-12-26

**Authors:** Manar K Almaliki, Ghidaa A Alghamdi, Refal M Mahrouqi, Rifal S Alsharif, Malak W Alsabban, Waref H Felemban, Mohammed H Ageel

**Affiliations:** 1 Medicine and Surgery, College of Medicine, Umm Al-Qura University, Makkah, SAU; 2 Pediatric Surgery, College of Medicine, Umm Al-Qura University, Makkah, SAU

**Keywords:** awareness, childhood, congenital conditions, congenital hernia, inguinal hernia, knowledge, online questionnaire, parental perception

## Abstract

Introduction

Congenital inguinal and umbilical hernias are common in pediatric populations and may lead to serious complications if not identified and managed promptly. Limited parental awareness can delay diagnosis and treatment, resulting in adverse health outcomes.

Methods

A descriptive cross-sectional study was conducted using an online questionnaire distributed to parents residing in the Makkah region of Saudi Arabia. The survey assessed demographic characteristics, awareness, and knowledge related to the causes, symptoms, risks, and management of congenital inguinal and umbilical hernias. A total of 470 responses were included in the analysis.

Results

Most participants demonstrated poor knowledge regarding both types of hernias, with the majority unaware of common symptoms or potential complications. Female parents, those with a family history of hernias, and those who had previously noticed swelling in their children showed significantly higher awareness levels (p < 0.05).

Conclusion

There is a substantial lack of parental awareness regarding congenital inguinal and umbilical hernias and their associated complications. Targeted health education initiatives are needed to enhance awareness, promote early recognition, and support timely medical intervention for affected children.

## Introduction

Congenital inguinal and umbilical hernias are common surgical conditions encountered in the pediatric population, with varying degrees of clinical significance. Inguinal hernias occur when abdominal contents, typically a portion of the intestine, protrude through a defect in the inguinal canal. In contrast, umbilical hernias arise from a defect in the abdominal wall at the site of the umbilicus. According to recent studies, inguinal hernias affect approximately 1-5% of infants, with a higher prevalence in premature infants and those with low birth weight [1]. Umbilical hernias are also prevalent, particularly among infants, with an incidence of 10-25%, and are more common in children of African descent [2].

Awareness and understanding of these congenital conditions are crucial for early recognition and timely intervention. Despite their frequency, many parents and caregivers lack adequate knowledge of the signs and potential complications associated with hernias. A study by Assakran et al. reported that a significant portion of parents were unaware of the possible risks of untreated hernias, such as incarceration and strangulation, which can lead to serious morbidity and even mortality [3]. This lack of awareness may contribute to delayed diagnosis and treatment, underscoring the need for improved education among both healthcare providers and the public.

The primary management of congenital inguinal and umbilical hernias involves surgical intervention, which is generally safe and effective. However, clinical approaches vary, and the timing of surgery can significantly impact patient outcomes. Umbilical hernias, in particular, may be monitored for spontaneous closure in cases where the defect is small and asymptomatic. One study found that by the age of five, approximately 88.6% of children experienced spontaneous closure of umbilical hernias [4].

Complications associated with congenital hernias can be severe. Incarceration occurs when herniated tissue becomes trapped, compromising its blood supply, while strangulation can result in tissue necrosis if not addressed promptly. These complications may present as testicular atrophy, hernia recurrence, or bowel ischemia [5]. The overall complication rate for umbilical hernias in infants is estimated to be around 2-4% [6], while the complication rate for congenital inguinal hernias is estimated at approximately 6.5% [7].

The potential for these serious outcomes highlights the necessity for healthcare professionals to educate families about the signs of hernias and the importance of seeking timely medical advice. Therefore, this study aims to evaluate parental awareness of congenital inguinal and umbilical hernias and their potential complications in the Makkah region of Saudi Arabia.

## Materials and methods

The study adopted a web-based, descriptive cross-sectional design, which is suitable for assessing knowledge and awareness within a specific population at a single point in time. Data were collected through an online questionnaire targeting parents of pediatric patients residing in the Makkah region of Saudi Arabia. This design was chosen to facilitate wide participation and accessibility, allowing parents to complete the questionnaire conveniently through a secure online platform. The objective was to assess parental knowledge and awareness regarding congenital inguinal and umbilical hernias, including definitions, risk factors, complications, and management.

The study was conducted over a 12-month period, including data collection, analysis, and manuscript preparation. The study was approved by the Institutional Review Board (IRB) of Umm Al-Qura University (approval number: HAPO-02-K-012-2025-10-2953). Participation was voluntary, and informed consent was obtained electronically at the beginning of the questionnaire. No personally identifying information was collected, ensuring that all responses remained anonymous. Data was securely stored in a password-protected format, accessible only to the research team.

Study population

The study aimed to include all parents living in the Makkah region with at least one child. Parents who were not residents of Makkah, those without internet access, and individuals who did not have children were excluded. The required sample size was calculated for a cross-sectional design, assuming a 95% confidence level, targeting a minimum of 385 participants through a convenience sampling method.

Study tool

A simple, succinct questionnaire was designed using Google Forms (Google LLC, Mountain View, California, United States), and participants received an electronic link with a brief explanation of the study's objectives and a request for voluntary participation, distributed via data collectors to the eligible population. The online questionnaire, which was developed and validated by the authors of a previous study [8], was divided into two sections: the first collected demographic data such as gender, residency, marital status, educational level, and number of children, while the second assessed general awareness about umbilical and inguinal hernias. The questionnaire included multiple-choice and true/false questions to gather comprehensive information about parents' understanding and perceptions of these conditions. Knowledge scoring was based on participants’ correct responses, with a total score converted to a percentage. A 60% cutoff point was used to distinguish between adequate and inadequate knowledge levels, consistent with prior studies [9,10].

Data analysis

Data analysis was performed using IBM SPSS Statistics for Windows, version 28.0 (Released 2021; IBM Corp., Armonk, New York, United States). Descriptive statistics were used to summarize the socio-demographic characteristics of the study participants, including frequency and percentage distributions for categorical variables. To assess parents' knowledge regarding congenital inguinal and umbilical hernias and their complications, each correct response was awarded one point, and the total knowledge score was calculated by summing the individual scores. Parents with a total score below 60% were categorized as having poor knowledge, while those with scores equal to or above 60% were classified as having good knowledge. Inferential statistical analyses were conducted to examine associations between overall knowledge level and independent variables. The Pearson Chi-square test was applied to assess relationships between categorical variables, such as gender, marital status, number of children, educational level, and family history of hernia. In cases where expected cell frequencies were less than five, Fisher’s exact test was used to ensure statistical validity. A p-value of less than 0.05 was considered statistically significant. 

## Results

Table 1 presents the socio-demographic characteristics of the study population. Of the 470 participants, the majority were female (n=343, 73.0%). In terms of marital status, a significant proportion of participants were married (n=318, 67.7%). Regarding the number of children, the largest group had four or more children (n=160, 34.0%), followed by those with one child (n=108, 23.0%), two children (n=105, 22.3%), and three children (n=97, 20.6%). Educationally, most participants had a bachelor’s degree (n=180, 38.3%). Regarding the symptoms observed in their children between one month and 12 years, the majority reported no symptoms (n=333, 70.9%), while 51 (10.9%) noticed umbilical swelling, 54 (11.5%) inguinal swelling, and 32 (6.8%) reported both swellings. As for the family history of hernias, most participants reported no history of umbilical or inguinal hernia (n=305, 64.9%).

**Table 1:** Socio-demographic characteristics of the study participants (N=470)

Data	Frequency	Percentage
Gender		
Male	127	27.0%
Female	343	73.0%
Marital status		
Married	318	67.7%
Divorced/widowed	152	32.3%
Number of children		
1 child	108	23.0%
2 children	105	22.3%
3 children	97	20.6%
4 or more children	160	34.0%
Educational level		
Intermediate school	33	7.0%
Secondary school	94	20.0%
Diploma	70	14.9%
Bachelor degree	180	38.3%
Postgraduate degree	93	19.8%
Have you noticed any of the following symptoms in your child between the ages of one month and 12 years?		
Nothing	333	70.9%
Umbilical swelling	51	10.9%
Inguinal swelling	54	11.5%
Both swellings	32	6.8%
Family history of umbilical/inguinal hernia		
None	305	64.9%
Umbilical hernia	90	19.1%
Inguinal hernia	42	8.9%
Both of them	33	7.0%

Table 2 outlines the knowledge and awareness of congenital inguinal hernia and its complications among parents in the Makkah Region, Saudi Arabia. Regarding the recognition of this condition, 33.2% (n=156) of parents knew only about umbilical hernias, 8.9% (n=42) knew only about inguinal hernias, 18.5% (n=87) were aware of both types, and 39.4% (n=185) had no knowledge of either. When asked about the usual solution for simple inguinal hernia, 34.9% (n=164) considered it a moderate condition that needed medical consultation, while 12.1% (n=57) thought it did not require a doctor’s visit. A significant portion of parents (n=191, 40.8%) believed that both types of hernias could recur after treatment, while 31.0% (n=145) disagreed. Regarding the contents of an inguinal hernia bulge, 21.6% (n=101) correctly identified part of the intestine, while 41.7% (n=195) did not know. A majority of parents (n=249, 53.2%) recognized a link between undescended testicles and inguinal hernias, while 46.8% (n=219) did not. Lastly, when asked to rate their knowledge of this hernia, a large majority (n=361, 77.1%) considered their knowledge limited, while 19.9% (n=93) rated it intermediate, and only 3.0% (n=14) felt they had good knowledge.

**Table 2:** Knowledge and awareness of the study participants regarding congenital inguinal hernia and its complications in the pediatric population

Knowledge items	Frequency	Percentage
Do you know what an umbilical hernia or congenital inguinal hernia is?		
Only an umbilical hernia	156	33.20%
Only inguinal hernia	42	8.90%
Both of them	87	18.50%
I don't know	185	39.40%
In most simple umbilical hernia cases, what is the usual solution?		
A simple condition that does not require a visit to the doctor	79	16.80%
Simple condition, but it's better to see a doctor	189	40.20%
Moderate condition and need to see a doctor	116	24.70%
Serious condition requiring urgent medical intervention	86	18.30%
In most simple inguinal hernia cases, what is the usual solution?		
A simple condition that does not require a visit to the doctor	57	12.10%
Simple condition, but it's better to see a doctor	149	31.70%
Moderate condition and need to see a doctor	164	34.90%
Serious condition requiring urgent medical intervention	100	21.30%
Do you think umbilical and inguinal hernias can recur after treatment?		
Yes, both types	191	40.80%
Umbilical hernia only	67	14.30%
Inguinal hernia only	65	13.90%
None	145	31.00%
The most common thing found inside an inguinal hernia bulge		
Testicle for males	66	14.10%
Ovaries for females	40	8.50%
Part of the intestine	101	21.60%
Fluids	44	9.40%
Air	22	4.70%
Don't know	195	41.70%
The most common thing found inside an umbilical hernia bulge		
Testicle for males	36	7.70%
Ovaries for females	35	7.50%
Part of the intestine	139	29.70%
Fluids	52	11.10%
Air	41	8.80%
Don't know	165	35.30%
There is a relationship between undescended testicles in children and inguinal hernia		
Yes	249	53.20%
No	219	46.80%
How would you rate your level of knowledge about umbilical and congenital inguinal hernias?		
Limited	361	77.10%
Intermediate	93	19.90%
Good	14	3.00%

**Figure 1:**
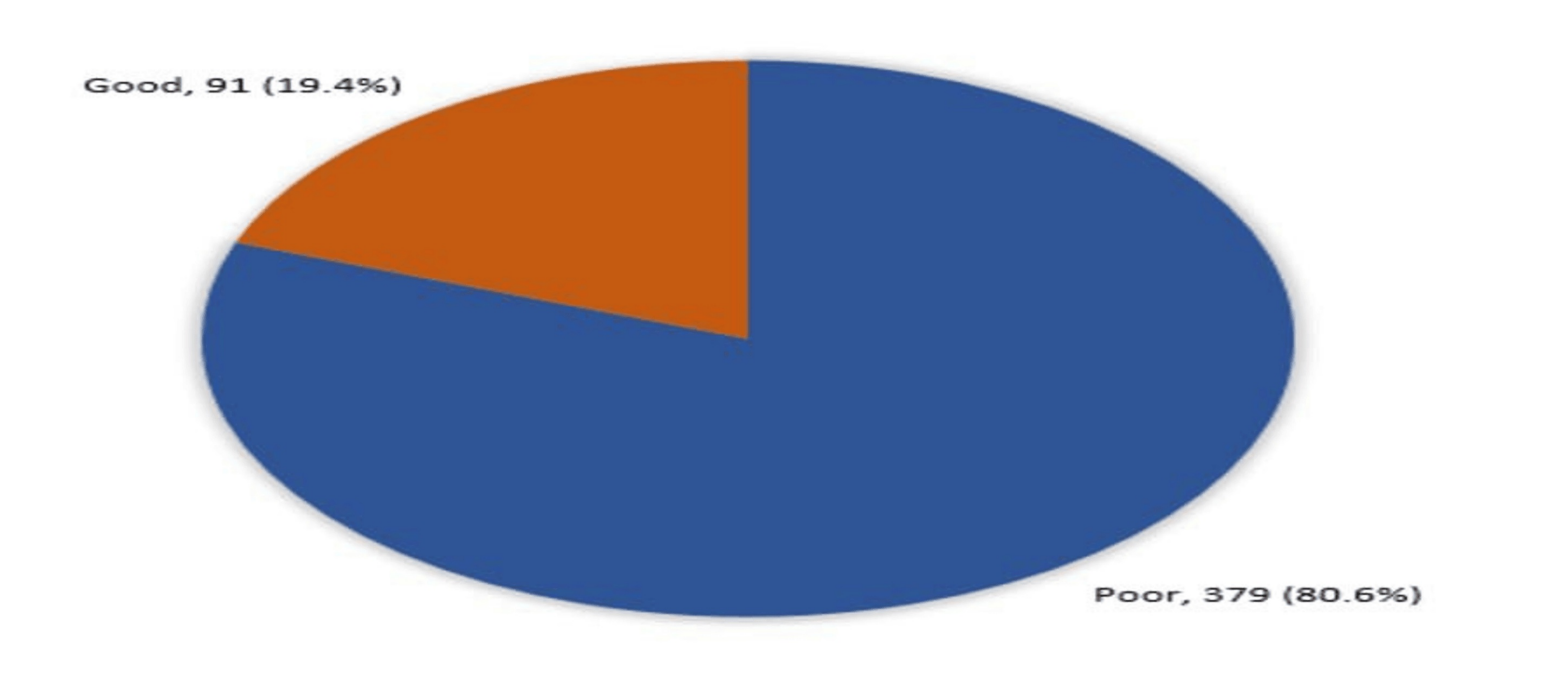
The overall knowledge and awareness of the study participants regarding congenital inguinal hernia and its complications in the pediatric population

Figure 1 shows the overall knowledge and awareness levels of congenital inguinal hernia and its complication among parents in the Makkah Region. The majority of participants (n=379, 80.6%) had a poor level of knowledge, while only 19.4% (n=91) showed a good understanding of the condition.

Table 3 examines the association between various socio-demographic and clinical factors and the overall knowledge level of congenital inguinal hernia among parents in the Makkah Region, Saudi Arabia. A statistically significant association was found between gender and knowledge level (p = 0.048), with a higher proportion of female parents (21.3%) having good knowledge compared to males (14.2%). Additionally, noticing symptoms in children was significantly associated with knowledge (p = 0.049); parents who observed both umbilical and inguinal swellings had the highest level of good knowledge (31.3%), compared to only 17.4% among those who noticed no symptoms. Family history of hernia also showed a significant relationship (p = 0.026), where 33.3% of those with a family history of inguinal hernia and 30.3% with both types reported good knowledge, compared to just 17.0% among those with no family history. On the other hand, marital status (p = 0.521), number of children (p = 0.563), and educational level (p = 0.939) were not significantly associated with knowledge level.

**Table 3:** Association between socio-demographic and clinical factors and overall knowledge level of congenital inguinal hernia among study participants (N=470) P: Pearson X2 test; P < 0.05 (significant)

Factors	Overall knowledge level				p-value
	Poor		Good		
	Frequency	Percentage	Frequency	Percentage	
Gender					.048*
Male	109	85.80%	18	14.20%	
Female	270	78.70%	73	21.30%	
Marital status					0.521
Married	259	81.40%	59	18.60%	
Divorced/widowed	120	78.90%	32	21.10%	
Number of children					.563^
1 child	87	80.60%	21	19.40%	
2 children	83	79.00%	22	21.00%	
3 children	83	85.60%	14	14.40%	
4/ more children	126	78.80%	34	21.30%	
Educational level					0.939
Intermediate school	25	75.80%	8	24.20%	
Secondary school	75	79.80%	19	20.20%	
Diplome	58	82.90%	12	17.10%	
Bachelor degree	146	81.10%	34	18.90%	
Post-graduate degree	75	80.60%	18	19.40%	
Have you noticed any of the following symptoms in your child between the ages of 1 month and 12 years?					.049*
Nothing	275	82.60%	58	17.40%	
Umbilical swelling	42	82.40%	9	17.60%	
Inguinal swelling	40	74.10%	14	25.90%	
Both swellings	22	68.80%	10	31.30%	
Family history of umbilical/inguinal hernia					.026*
None	253	83.00%	52	17.00%	
Umbilical hernia	75	83.30%	15	16.70%	
Inguinal hernia	28	66.70%	14	33.30%	
Both of them	23	69.70%	10	30.30%	

Table 4 explores the association between family history of inguinal hernia and the observation of related symptoms in children aged one month to 12 years. The association was statistically significant (p = .001), indicating that parents with a family history of hernia were more likely to notice symptoms in their children. Among parents with no family history, the majority (84.6%) reported no symptoms in their children, while very few noticed umbilical swelling (5.2%), inguinal swelling (5.9%), or both swellings (4.3%). In contrast, among those with a family history of an inguinal hernia, 50.0% observed inguinal swelling and 11.9% reported both types of swelling. Notably, 30.3% of parents with a history of both hernia types noticed both swellings in their children, compared to only 4.3% in the group with no family history.

**Table 4:** Association between family history of hernia and noticing symptoms in children aged one month to 12 years P: Exact probability test; P < 0.05 (significant)

Family history of umbilical/inguinal hernia	Have you noticed any of the following symptoms in your child between the ages of 1 month and 12 years?								p-value
	Nothing		Umbilical swelling		Inguinal swelling		Both swellings		
	Frequency	Percentage	Frequency	Percentage	Frequency	Percentage	Frequency	Percentage	
None	258	84.60%	16	5.20%	18	5.90%	13	4.30%	.001*^
Umbilical hernia	50	55.60%	28	31.10%	8	8.90%	4	4.40%	
Inguinal hernia	13	31.00%	3	7.10%	21	50.00%	5	11.90%	
Both of them	12	36.40%	4	12.10%	7	21.20%	10	30.30%	

The multivariable analysis (Table 5) showed that most demographic factors were not significantly associated with parental awareness of congenital inguinal and umbilical hernias. Marital status, number of children, and educational level had no significant effect on awareness. In contrast, two factors were reported as significant predictors. Female parents had higher odds of being aware compared with males, with the association reaching statistical significance (adjusted odds (AOR) = 1.81; 95%CI: 1.00-3.30; p = 0.049). Additionally, parents with a family history of umbilical or inguinal hernias were more likely to be aware of these conditions (AOR = 1.34; 95%CI: 1.06-1.70; p = 0.016).

**Table 5:** Multivariable logistic regression analysis of predictors of parental awareness of congenital inguinal and umbilical hernias AOR: adjusted odds ratio; CI: confidence interval; P < 0.05 (significant)

Predictors	p-value	OR_A_	95% CI	
			Lower	Upper
Unmarried vs. Married	0.482	1.22	0.71	2.09
Number of children	0.878	1.02	0.82	1.26
Higher educational level	0.899	0.99	0.81	1.2
Female vs. male gender	0.049*	1.81	1	3.3
Family history of umbilical/inguinal hernia	0.016*	1.34	1.06	1.7

## Discussion

The study population consisted predominantly of female parents. A notable proportion of the participants were married, with the largest segment having four or more children. In terms of educational level, the majority of the parents held a bachelor's degree. Regarding their children within the specified age range, most parents observed no symptoms indicative of a hernia. Among those who did notice symptoms, umbilical and inguinal swellings were the primary observations, with a smaller group reporting both. The majority of the participating parents indicated no family history of either umbilical or inguinal hernias, although a considerable portion reported a history of umbilical hernia in their families, followed by a smaller percentage with a family history of inguinal hernia or both types.

Regarding hernia awareness among parents, the current study showed a significant defect in their knowledge and awareness regarding congenital inguinal and umbilical hernias. The results showed that a substantial proportion of parents lack basic understanding of these conditions, their complications, and appropriate management strategies.

Knowledge of congenital inguinal hernia

In this study, only a few parents were aware of both inguinal and umbilical hernias, while more than one-third did not know about either condition. This lack of awareness is concerning, given that congenital hernias are common in pediatric populations, with inguinal hernias occurring in 1-5% of children and umbilical hernias in 10-20% of infants [11]. A similar study conducted in Nigeria found that 47.2% of parents had heard about umbilical hernias, but only 28.6% were aware of inguinal hernias [12]. Comparatively, a study in Saudi Arabia showed that awareness of both umbilical and inguinal hernias was limited to about one-fifth of the participants. The overall knowledge regarding hernias was low, with the majority demonstrating poor knowledge [3]. Several studies documented a lack of knowledge about adult hernias among the general population [13-18]. However, this was contradicted by the study by Mafouz et al. (2020) [19], Alsaigh et al. (2023) [20], and Al Judia et al. (2018) [21], which indicates adequate hernia knowledge in the adult population.

Perceptions of hernia severity and need for medical care

Regarding management, less than half of the parents in our study believed that umbilical hernias required a doctor’s consultation, while a small percentage considered them harmless and did not need medical attention. This contrasts with a study from Egypt, where 58% of parents sought medical advice for umbilical hernias, reflecting higher health-seeking behavior [22]. The misconception that umbilical hernias are trivial may originate from cultural beliefs that they resolve spontaneously, which is true in many cases but not all [23]. For inguinal hernias, one-third of parents in our study recognized them as moderate conditions requiring medical attention, while a few of them underestimated their severity. This is concerning because inguinal hernias in children carry a risk of incarceration and strangulation, requiring prompt surgical intervention [24].

Understanding of hernia contents and complications

Only one-fifth of parents correctly identified intestinal contents in an inguinal hernia, while 29.7% did so for umbilical hernias. This indicates a poor understanding of hernia pathophysiology, which aligns with findings from a Pakistani study where only 18.5% of parents knew about hernia contents [25]. A notable finding was that about half of the parents reported an association between undescended testicles and inguinal hernias. However, nearly half of the parents in our study remained unaware of this link, which is clinically significant since cryptorchidism increases hernia risk [26]. Generally, the vast majority of parents rated their knowledge as limited, with only 3% believing they had a good understanding. This matches the assessed overall knowledge level, where 80.6% had poor knowledge levels, and also the poor level of parental knowledge locally [3,27] and internationally.

The study identified several socio-demographic and clinical factors influencing parental knowledge of congenital hernias. Female parents showed a higher level of knowledge compared to their male counterparts, a finding consistent with research indicating mothers' greater involvement in child healthcare. Experiencing a child with both umbilical and inguinal swellings was associated with the highest knowledge scores, highlighting the impact of direct observation on awareness. Furthermore, a family history of hernias, particularly inguinal or both types, strongly correlated with increased parental knowledge, suggesting familial experience plays a role in recognition. Interestingly, factors such as education level, marital status, and the number of children did not significantly influence knowledge. However, this finding should be interpreted with caution, as the study sample predominantly consisted of highly educated parents, which may have limited the variability needed to detect such associations.

Limitations

This study has several limitations. First, the use of a convenience sampling method and an online questionnaire may limit the generalizability of the findings, as participants with internet access and higher education levels were likely overrepresented, whereas parents with limited literacy or restricted internet access may have been underrepresented. Second, the cross-sectional design captures awareness at a single point in time, preventing the evaluation of causal relationships. Third, self-reported data may be subject to recall bias or social desirability bias. Finally, the study focused exclusively on parents residing in the Makkah region, which may not accurately reflect awareness levels in other regions of Saudi Arabia.

## Conclusions

This study reveals a considerable lack of knowledge among parents in the Makkah region concerning congenital inguinal and umbilical hernias, with only a small fraction showing good awareness. Being of the female sex, having previously noticed hernia symptoms in a child, and having a family history of hernias were linked to better knowledge. Surprisingly, education level and marital status didn't seem to make a difference. However, this finding should be interpreted cautiously, as the sample predominantly consisted of highly educated parents, which may have limited the ability to detect associations with education level. When we compare these findings to studies in other countries, ‏It appears that awareness levels among parents in Makkah region may be relatively lower compared to other regions, which could be associated with limited health education initiatives. Physicians and nurses should talk to parents about hernias during regular checkups, and we should have educational programs in local communities to help people recognize the signs early and seek medical help when needed. Furthermore, using social media and information from healthcare centers can be a great way to spread accurate information.

## Appendices

 Study questionnaire

**Table 6:** Section One: Sociodemographic Characteristics of the Parents

Gender	
Male	
Female	
Marital Status	
Married	
Divorced	
Widowed	
Number of Children	
None	
One	
Two	
Three	
Four or more	
Educational Level	
Middle school	
Secondary school	
Diploma holder	
Bachelor’s degree	
Master’s degree	
PhD	

**Table 7:** Section Two: General Awareness about Congenital Inguinal or Congenital Umbilical Hernia

Do you know what congenital inguinal or congenital umbilical hernia is						
Umbilical hernia only						
Inguinal hernia only						
Both umbilical and inguinal hernias						
None of the above						
Have you noticed any of your children exhibiting any of the following symptoms when they were between 1 month and 12 years old						
							Swelling in the umbilical area						
Swelling in the inguinal area						
Both						
None of the above						
Are there any of your family members who suffered from congenital umbilical hernia and/or inguinal hernias when he/she was between 1 month and 12 years old						
Umbilical hernia only						
Inguinal hernia only						
Both umbilical and inguinal hernias						
None of the above						

**Table 8:** Section Three: Assessment of Knowledge toward Congenital Inguinal or Congenital Umbilical Hernia

In most uncomplicated cases of umbilical hernia, what is the usual outcome							
It is a mild condition and does not require a visit to the doctor							
It is a mild condition but requires a visit to the doctor							
It is a moderate condition and requires a visit to the doctor							
This is a serious condition that requires emergency medical intervention							
In most uncomplicated cases of inguinal hernia, what is the usual outcome							
It is a mild condition and does not require a visit to the doctor							
It is a mild condition but requires a visit to the doctor							
It is a moderate condition and requires a visit to the doctor							
This is a serious condition that requires emergency medical intervention							
Do you think that congenital umbilical and inguinal hernias can recur after treatment							
Yes, umbilical hernia only							
Yes, inguinal hernia only							
Yes, both umbilical and inguinal hernias							
No							
In your opinion, what is the most common content found inside the bulge of the inguinal hernia							
Part of the intestine							
Ovaries (in females)							
Testicle (in males)							
Air							
Liquids							
I don’t know							
In your opinion, what is the most common content found inside the bulge of the umbilical hernia							
Part of the intestine							
Ovaries (in females)							
Air							
Liquids							
I don’t know							
Do you think there is a relationship between undescended testicles in children and inguinal hernia							
yes							
No							
How would you rate your level of knowledge about congenital umbilical and inguinal hernias							
Minimal (You have little to no information about the topic)							
Moderate (You have some knowledge but need further clarification)							
Comprehensive (You have a thorough understanding of the topic and its stages)							
